# 

**Published:** 1875-10

**Authors:** 


**Dr. Hall's Picture.**—The likeness of Dr. Hall, of the *Journal*, which appears on the outside, was drawn and engraved by Leggo Brothers & Co., *Times* Building, Park Row, New York. It will be recognized as a very good likeness, and was made in less than two days. The process is that of pen-drawing in large, and reducing by photographic process. The metal plate is then produced by a peculiar process, and can be printed from on a common press. In this art Leggo Brothers stand at the head, and we cordially recommend them to all requiring this class of illustration.

### HALL'S JOURNAL FOR 1876.

We desire and expect to put Hall's Journal of Health for next year into several thousand families which have not received its regular visits in 1875. To do this we propose to make an offer of an exceptionally attractive character. We therefore ask every one to

### READ THIS PARAGRAPH.

Nearly everybody takes or buys some newspaper or magazine—daily, weekly, or monthly—regularly. If any do not, they should. Now we wish to show all newspaper and magazine readers how they can take their favorite publication, at the regular subscription price, and at the same time become a subscriber to Hall's Journal of Health. We do not say that for the yearly price of the publication selected we will send both for an entire year; but we do agree to send the publication subscribed for, and also to send our journal for such period as one quarter part of the money sent would pay for at the regular rate of two dollars a year.

For example, Col. Fairbanks, of St. Johnsbury, Vermont, desires "Harper's Monthly Magazine" for 1876. The subscription price is $4. He remits us this sum, and we cause "Harper" to be mailed to him for one year, direct from the office of publication. One quarter of the $4 would be $1. This would pay for Hall's Journal of Health for six months; and therefore Col. Fairbanks is put down as a six months' subscriber to the Journal, and his $4 pays for the whole.

Take the case of Mr. O. F. Barron, proprietor of the "Junction House," at White River, Vermont, and the "Crawford" and "Twin-Mountain" Houses, at the White Mountains. He wants "Godey's Lady's Book" ($3) and the "Weekly Tribune" ($2), total $5. We cause his name to be entered for both these publications, and also for our Journal for the value of $1.25,—one quarter of all the money remitted. He gets both the publications subscribed for one year, and the Journal eight months for nothing.

This is our new plan, and it ought to work well. It is not difficult to understand. We simply give the value of all the money sent, and one fourth of said value in subscription to the Journal besides. For $1 we give $1.25; for $2 we give $2.50; for $3 we give $3.75; for $4 we give $5; for $5 we give $6.25; for $6 we give $7.50; for $7 we give $8.75; and for $8 we give $10. We make this offer with reference to any book, not rare and out of print, as well as in regard to papers and magazines; and we include English publications as well. The commissions allowed us enable us to do this. So select your book or paper, and, if it can be bought, we will supply it, and add the Journal to the value of one quarter of all money received.

**Offer to Subscribers.**—The sum of $2, received by us during the month of October, as a subscription to Hall's Journal of Health for 1876, will entitle the subscriber to the three closing numbers of 1875 without cost. A like sum reaching us in November, secures fourteen monthly numbers; and if received in December, pays for thirteen numbers.

**Our Twenty-Third Year.**—We shall introduce some new and valuable features into our twenty-third volume. Among other matters of interest to the thoughtful, we hope to devote a good deal of attention to such health, sanitary, hygienic, and food topics, as are illustrated by articles on exhibition at our Centennial Exposition in Philadelphia.

### THE HEALTH FOOD COMPANY.

This company is certainly doing a thriving business in the sale of the "Granulated Wheat Biscuit. This is an excellent food, building up the waste places of humanity very rapidly, and affording solid nourishment of a palatable kind. We have used the Granulated Wheat Biscuit for some months, and confess that it agrees with us better than any bread we have yet found. Its effect upon the stomach and bowels is very salutary, and the mode of preparation fits it for very easy digestion. Besides, it is a comfort to know, as we do in this instance, that it is a positively clean food, free from dust and smut and dirt, and all foreign substances. The old and the young, the feeble and the strong, find in this clean, well-cooked, and wholesome bread, the very best single food to be had anywhere. Whenever superfine flour shall be discarded, and food like this used instead, we shall see human beings with rosier cheeks, better complexions, less angular forms, better teeth, stronger constitutions, more powerful brains, and longer lives. A generation of men and women fed largely on these honest, whole-wheat foods, would be a wonderful people indeed. We propose to keep preaching, and hope to bring the nation up to something like a worthy standard, by and by. But it must abandon starch as a leading article of food, before it ever attains to its true grandeur.

The Health Food Company is our nearest neighbor, at 137 Eighth Street, New York, and we are glad to vouch for its high character and its earnest devotion to the good of mankind. If postage-stamps are sent them, they send samples of their biscuit by mail.

